# Exploring salinity-induced biochemical changes in *Chlorella vulgaris* using statistical modelling

**DOI:** 10.1038/s41598-025-11110-x

**Published:** 2025-10-21

**Authors:** Ana F. Esteves, Ana L. Gonçalves, Vítor J. P. Vilar, José C. M. Pires

**Affiliations:** 1 LEPABE – Laboratory for Process Engineering, Environment, Biotechnology and Energy, Faculty of Engineering, University of Porto, Rua Dr. Roberto Frias, 4200-465 Porto, Portugal; 2 ALiCE – Associate Laboratory in Chemical Engineering, Faculty of Engineering, University of Porto, Rua Dr. Roberto Frias, 4200-465 Porto, Portugal; 3 LSRE-LCM – Laboratory of Separation and Reaction Engineering-Laboratory of Catalysis and Materials, Faculty of Engineering, University of Porto, Rua Dr. Roberto Frias, 4200-465 Porto, Portugal; 4 CITEVE – Technological Centre for the Textile and Clothing Industries of Portugal, Rua Fernando Mesquita, 2785, 4760-034 Vila Nova de Famalicão, Portugal

**Keywords:** Microalgal biomass composition, Multiple linear regression, Principal component analysis, Salt stress, Two-stage cultivation, Biotechnology, Plant sciences, Environmental sciences

## Abstract

The impact of high salinity stress on the growth and nutrient uptake of *Chlorella vulgaris* was investigated using a two-stage cultivation approach. Changes in the biochemical composition of the biomass were statistically evaluated through principal component analysis (PCA) and multiple linear regression (MLR). The presence of 150 mM NaCl promoted higher biomass accumulation (978 ± 11 mg L^−1^) and did not affect the nutrient removal rates. Elevated concentrations (> 300 mM) induced negative effects, resulting in microalgae mortality. The highest lipid (24% ± 1% DCW) and carbohydrate (32.3% ± 0.6% DCW) contents were achieved with 300 mM NaCl on days 7 and 4, respectively. Moreover, 150 mM NaCl led to the highest lipid productivity (23.4 mg L^−1^ d^−1^) only in 2 days of stress. PCA and MLR confirmed that the lipid content was positively associated with salinity and time of exposure and that the pigments were strongly affected by the exposure time.

**Supplementary Information:**

The online version contains supplementary material available at 10.1038/s41598-025-11110-x.

## Introduction

Microalgae have attracted increasing interest due to their diverse applications in biotechnology, renewable energy production and wastewater treatment. These photosynthetic microorganisms exhibit several advantages, including rapid growth rates, the ability to capture CO_2_ (with higher efficiency compared with terrestrial plants), treat wastewater, grow under harsh conditions, and accumulate different types of compounds in their biomass, such as lipids, carbohydrates, proteins, and pigments.

Microalgae are affected by diverse environmental conditions, such as salinity. This operational parameter has a great influence on the cell membrane permeability, osmoregulation, enzyme activities and metabolic processes^1^. Since this parameter affects the osmotic pressure of the cell, consequently, the transfer of carbon and other nutrients across the cell membrane is also affected^2^. Overall, microalgae are highly adaptable to varying levels of salinity. However, high salt concentration can lead to the destruction of cellular structures essential to photosynthetic processes, the increase of reactive oxygen species (ROS) and consequently cause oxidative stress, lower biomass productivity and even cellular disruption^3^. To diminish the effects of salinity stress, microalgae produce antioxidant compounds and accumulate energy source compounds (e.g., lipids), which are considered valuable feedstock in multiple industries^4^. In addition to increasing the production of lipids and antioxidant compounds, adding salt or salt water to the microalgal culture medium also brings other advantages, such as reducing the risk of microbial contamination and using less freshwater in microalgal cultivation^5^. To solve the problem regarding the low productivity of compounds of interest in high-salinity environments, two-stage cultivation can be employed. In the first stage, microalgae are cultivated under optimal conditions, enhancing biomass productivity. Then, in the second stage, a stress condition is applied to increase the production of compounds of interest (such as lipids or carotenoids)^6,7^. Nezafatian et al.^8^ grew *Tetraselmis tetrathele* with the two-stage cultivation technique. In the second stage, the authors applied salinity stress (40 g L^−1^) and observed an increase in pigment production. Li et al.^9^ also performed a two-stage cultivation of *Haematococcus pluvialis* with NaCl as the stress factor. The authors reported that adding 2 g L^−1^ NaCl to the medium (corresponding to approximately 34 mM NaCl) led to improvements in lipid and astaxanthin production.

The majority of the studies from the literature regarding lipid production use nutrient starvation as a stress factor^10–12^, but the salinity stress can also be used with the same goal. Gour et al.^13^ tested the effect of salinity on the growth and lipid content of different halotolerant species (*Scenedesmus quadricauda* and *Scenedesmus dimorphus*). The authors observed an increase in microalgal growth up to the salinity level of 40 mM NaCl and a higher accumulation of lipids with NaCl concentration of 160 mM for all species. Maneechote and Cheirsilp^14^ applied different stress factors individually (salinity, light intensity and temperature) to *Scenedesmus* sp.. The highest biomass concentration and lipid content were achieved under 0.5% salinity, 4000 lux of light intensity and a temperature of 30 °C. You et al.^15^ observed that saline stress conditions induced the accumulation of lipid droplets in the marine microalga *Parachlorella kessleri*. Zhang et al.^16^ reported higher lipid content with increasing NaCl concentrations from 0 to 2% NaCl in *Chlamydomonas* sp.; however, the highest lipid productivity was achieved with 2% NaCl (approximately 349 mM NaCl). Ajayan et al.^17^ used a Box-Behnken Design to optimise the NaCl, nitrate, and phosphate concentrations used during *Kirchneriella obesa* cultivation. The authors demonstrated that optimised conditions (2.5 g L^−1^ nitrate, 0.04 g L^−1^ phosphate, and 25 mM NaCl) led to significantly higher lipid accumulation (46% DCW) and carotenoid content (2.58 mg L^−1^). Most studies regarding salinity only focus on lipid content in biodiesel production. However, it is essential to also analyse other compounds such as carbohydrates (which can also be used to produce biofuels) as well as proteins to understand the trade-offs and synergies between different biochemical pathways. By understanding the mechanisms behind the microalgal responses to salinity stress, it is possible to enhance salinity stress tolerance and further optimise cultivation strategies for improving biomass productivity and accumulating compounds of interest. The existent studies have primarily centred on the effect of higher salinity levels on marine species, overlooking other freshwater species that may hold significant potential. Furthermore, the use of freshwater species in saline environments presents a good opportunity to lessen their dependence on freshwater sources, reducing their use and contributing to diminishing the water scarcity concerns.

In this study, the effects of high salinity stress on the growth, nutrient uptake and biomass composition of *Chlorella vulgaris* were investigated. Specifically, the main goals of the study were: (i) evaluate the growth and microalgal productivity under varying salinity levels (0, 150, 300, 450 and 600 mM NaCl) with a two-stage cultivation technique; (ii) quantify the nutrient uptake during stress conditions; (iii) assess changes in the biochemical composition of the biomass throughout time in response to salinity stress; (iv) better understand the underlying mechanisms and the relationships between the biomass composition, salinity stress and exposure time through principal component analysis (PCA) and multiple linear regression (MLR). In the industry context, understanding how salinity stress affects microalgae biomass is essential to improve the efficiency and economic viability of the production of biofuels. Moreover, analysing the growth dynamics and nutrient uptake can contribute to the development of a sustainable and robust microalgae culture system, tailoring maximum productivity and compound accumulation. From a social perspective, the transition from fossil fuels to sustainable biofuels is essential for reducing greenhouse gas emissions and achieving an important goal regarding energy sustainability and security. By understanding how salinity affects growth, nutrient uptake and biomass composition, new scientific advances can be reached in the field of biofuel production and microalgae-based biotechnologies, leading to more sustainable processes.

## Materials and methods

### Microalgal species and cultivation conditions

The microalgal strain used in this study, *C. vulgaris* CCAP 211/11B, was provided by the Culture Collection of Algae and Protozoa (UK). The stock cultures were grown in a modified OECD medium^18^. These cultures were kept at room temperature, with a light intensity of 50 µmol m^−2^ s^−1^ and a 24:0 light/dark cycle. The microalgae were agitated by an orbital shaker (Unimax 1010, Heidolph, Germany) at 120 rpm.

### Two-stage cultivation setup

In the first stage of the cultivation, the cultures were grown under the modified OECD medium but with a doubled concentration of nutrients. This modification aimed to provide ample nutrient availability to support optimal growth conditions and minimise the potential for nutrient limitation during the second stage. The experiments were conducted in batch mode in 1-L flasks (in duplicate for each salt condition tested in the second stage). Daily measurements of temperature, pH and conductivity were conducted using an electrochemical analyser (C6010, Consort, Belgium). Throughout the experiment, the temperature was maintained at 21 ± 2 °C, while pH was regulated at 7 by injecting a pure CO_2_ stream into the culture medium. The culture was aerated by injecting filtered air (0.45-μm nylon membrane filters) at the bottom using an air pump (Sicce Airlight 3300, Italy) at a flow rate of 1.7 L min^−1^. This aeration process facilitated the homogenisation of the culture. White light-emitting diode (LED) light panels (35 W, 4000 K) were used as a light source. During the first stage, the culture grew with increasing light intensity, beginning with 291 µmol m^−2^ s^−1^ until reaching 1107 µmol m^−2^ s^−1^ and with a 24:0 light/dark ratio. In the second stage, the light intensity was kept constant at 1107 µmol m^−2^ s^−1^ with a 24:0 light/dark ratio. NaCl was used to induce the salinity stress, and it was added to the cultures on day 7. The salt effect was evaluated for 7 days. Two groups of salt stress assays were performed due to space issues. In the first group (stress group 1), three NaCl concentrations were tested: 0 (control 1), 150 and 300 mM. In the second group (stress group 2), additional NaCl concentrations were tested: 0 (control 2), 450 and 600 mM. The maximum salinity value used in the experiment is approximately equivalent to the salinity of seawater. All cultivations were carried out using the modified OECD medium.

### Biomass sampling and analysis

Daily samples were collected to assess the microalgal growth through optical density (OD) at 680 nm with the Spectroquant Prove 300 spectrophotometer (Merck, Germany). Equation (1) was used to calculate the biomass concentration ($X$) in dry weight (DCW) from the previously measured OD.

1 $${\text{X }}\left( {{\text{mg L}}^{ - 1} } \right) = \left( {175 \pm 7} \right) \times {\text{OD}} - \left( {12 \pm 7} \right);{\text{ R}}^{2} = 0.993$$

The specific growth rate ($\mu$) using Eq. (2):

2 $${\mu }\left( {{\text{d}}^{ - 1} } \right) = \frac{{{\text{ln}}\left( {{\text{X}}_{2} /{\text{X}}_{1} } \right)}}{{{\text{t}}_{2} - {\text{t}}_{1} }}$$

where $X_{1}$ and $X_{2}$ are the biomass concentration at the time $t_{1}$ and time $t_{2}$ (in days), respectively.

The daily biomass productivity ($BP_{d}$) between consecutive samples ($X_{z}$ and $X_{z + 1}$) was calculated through Eq. (3). The maximum value of the $BP_{d}$ corresponds to the maximum productivity ($BP_{max}$). Moreover, the inhibition percentage (*IP*) was calculated for each stress treatment through Eq. (4), where $X_{c}$ is the biomass concentration of the control assay and $X_{t}$ is the biomass concentration of the stressed cultures at the end of the stress treatment.

3 $${\text{BP}}_{{\text{d}}} \left( {{\text{mg L}}^{ - 1} {\text{ d}}^{ - 1} } \right) = \frac{{{\text{X}}_{{{\text{z}} + 1}} - {\text{X}}_{z} }}{{{\text{t}}_{{{\text{z}} + 1}} - {\text{t}}_{z} }}$$

4 $${\text{IP}}  = \frac{{{\text{X}}_{c} - {\text{X}}_{t} }}{{{\text{X}}_{c} }} \times 100$$

The maximum quantum efficiency of photosystem II ($F_{v} /F_{m}$) was also measured (in triplicate) on days 0, 1, 2, 4 and 7 of the stress phase with a pulse amplitude-modulated fluorimeter (Junior-PAM, H. Walz GmbH, Germany). The data acquisition and recording were conducted using the WinControl-3 software. After acclimating the samples to darkness for 15 min, an optical plastic fibre (1.5 mm in diameter and 10 mm long) was immersed in the sample. Blue LED light (460 nm) was used for measurement, actinic illumination and saturating pulses. A saturation pulse was applied to determine $F_{v} /F_{m}$.

### Nutrient removal

The nutrient concentration (nitrate-nitrogen, NO_3_-N, and phosphate-phosphorus, PO_4_-P) was analysed at the beginning and end of the first stage. During the stress phase, samples were taken in triplicate on days 0, 1, 2, 4 and 7 of each salinity condition. All the samples were centrifuged (4 000 rpm, 10 min) and stored at − 20 °C. NO_3_-N was analysed according to the protocol of Collos et al.^19^ and PO_4_-P determination was conducted with a Spectroquant Phosphate Kit Test (Merck, Germany). The removal rate ($RR$) of each nutrient was calculated with Eq. (5).

5 $${\text{RR }}\left( {{\text{mg L}}^{ - 1} {\text{ d}}^{ - 1} } \right) = \frac{{{\text{S}}_{1} - {\text{S}}_{2} }}{{{\text{t}}_{2} - {\text{t}}_{1} }}$$

where $S_{1}$ and $S_{2}$ are the nutrient concentrations (mg L^−1^) at the time $t_{1}$ and time $t_{2}$, respectively.

### Biomass biochemical composition

Microalgae samples were collected on days 0, 1, 2, 4 and 7 of the stress stage. Biomass analysis (conducted in triplicate) included the quantification of lipids, carbohydrates, proteins and photosynthetic pigments (including chlorophyll a, chlorophyll b and carotenoids). The collected biomass underwent centrifugation at 12 000 rpm for 10 min, freezing at − 80 °C, and lyophilisation before maceration. The methods used to extract and quantify these compounds were described in Esteves et al.^20^. Briefly, the lipids were extracted with a methanol/chloroform/water mixture and quantified gravimetrically, following a modified Bligh and Dyer^21^ method. The carbohydrates were extracted using sulphuric acid, followed by digestion, and were quantified by spectrophotometry (with sulphuric acid and a phenol solution), following protocols developed by Dubois et al.^22^ and Van Wychen and Laurens^23^. The proteins were extracted with NaOH and quantified using an adapted protocol based on Lowry et al.^24^, by spectrophotometry. The photosynthetic pigments were extracted with methanol 90% (v/v) and quantified through specific equations proposed by Lightenthaler^25^.

Additionally, the lipid productivity ($LP$) and carbohydrate productivity ($CP$) were also calculated through the following equation:

6 $${\text{LP or CP }}\left( {{\text{mg L}}^{ - 1} {\text{ d}}^{ - 1} } \right) = \frac{{\left( {{\text{X}}_{{\text{z}}} \times {\text{CC}}_{{\text{z}}} } \right) - \left( {{\text{X}}_{0} \times {\text{CC}}_{0} } \right)}}{{{\text{t}}_{{\text{z}}} - {\text{t}}_{0} }}$$

where $X_{z}$ and $X_{0}$ represent the biomass concentration in mg L^−1^ and $CC_{z}$ and $CC_{0}$ is the compound content (lipids or carbohydrates) in % DCW on day *z* of the second stage ($t_{z}$) and on day 0 of the second stage ($t_{0}$).

### Statistical analysis

The parameters presented in this study were described by their mean and standard deviation. Differences among the studied conditions were assessed using one-way ANOVA, followed by the Tukey test, with a significance level set at 0.05.

The influence of salinity and exposure time on biomass composition was assessed through principal component analysis (PCA). The loadings of the principal components (PC) were determined to identify patterns between the chosen variables: salinity, exposure time, lipid content, carbohydrate content, protein content, chlorophyll content and carotenoid content. This statistical methodology was applied to identify the related variables. Prior to its application, the data were Z-standardised to have a mean of zero and a standard deviation of one. PCA is a mathematical procedure used to remove collinearity between observed variables, creating new uncorrelated variables (principal components, PCs) and typically reducing the dimensionality of the multivariate problem. These new variables are linear combinations of the original ones. The first principal component (PC1) captures the largest fraction of the original data variance, with each subsequent component accounting for as much of the remaining variability as possible. The number of PCs was determined using the Kaiser criterion, selecting those with an eigenvalue greater than 1. PCA is performed by the eigenvalue decomposition of a data covariance matrix. The results are usually interpreted by analysing the rotated factor loadings of the principal components. These loadings, which represent the contribution of each original variable to the PCs, are obtained through varimax rotation. Varimax rotation is the most widely used orthogonal rotation in PCA because it simplifies the interpretation of results by making the loadings high or low. This is achieved by rigidly rotating the PC axes so that the variable projections (loadings) on each PC become more distinct^26^.

Multiple linear regression (MLR) was also performed to evaluate the impact of salt concentration and exposure time on the content of the studied biomass compounds. The quadratic model was formulated for each biomass compound content, $y$, as follows:

7 $${\text{y}} = {\upbeta }_{0} + {\upbeta }_{1} {\text{x}}_{1} + {\upbeta }_{2} {\text{x}}_{2} + {\upbeta }_{11} {\text{x}}_{1}^{2} + {\upbeta }_{22} {\text{x}}_{1}^{2} + {\upbeta }_{12} {\text{x}}_{1} {\text{x}}_{2} + {\text{error}}$$

$\beta_{0}$ is the intercept, $\beta_{1}$ and $\beta_{2}$ are the linear coefficients for exposure time ($x_{1}$) and salinity ($x_{2}$), $\beta_{11}$ and $\beta_{22}$ represent the quadratic coefficients and $\beta_{12}$ is the interaction coefficient. The MLR models were developed using subroutines in Visual Basic for Applications for Microsoft Excel, created by the authors. The statistical significance of the regression parameters was assessed using a t-test with a significance level of 0.05. The final models were obtained by evaluating all combinations of input variables and selecting the best model, defined as the one with the lowest sum of squared errors, with the additional constraint that all regression parameters must be statistically significant^27^.

## Results and discussion

### Growth and productivity analysis (first stage)

In the first stage, the microalgae were grown under optimal conditions. The nutrient concentration in the medium was doubled to ensure that nutrient depletion did not happen in the second stage. Moreover, the light intensity gradually increased from 291 to 1107 µmol m^−2^ s^−1^ since it was the light intensity with the highest growth rate and productivity found in the literature for *C. vulgaris*^20^. During this phase, the microalgae achieved growth rates between 0.54 ± 0.01 d^−1^ and 0.59 ± 0.01 d^−1^ and ${BP}_{max}$ values ranging from 172 to 192 mg L^−1^ d^−1^ in group 1. In group 2, the specific growth rates reached values of 0.61 ± 0.01 d^−1^ and 0.62 ± 0.01 d^−1^, and the ${BP}_{max}$ values were between 118 and 122 mg L^−1^ d^−1^. Regarding nutrient uptake, both nitrogen and phosphorus were consumed by the microalgae. The NO_3_-N removal rates ranged between 4.3–5.0 mg L^−1^ d^−1^ in group 1 and between 3.1 and 3.4 mg L^−1^ d^−1^ in group 2. The PO_4_-P removal rates varied from 1.54 to 1.61 mg L^−1^ d^−1^ in group 1 and from 0.95 to 1.30 mg L^−1^ d^−1^ in group 2. These results are in accordance with the literature. Esteves et al.^20^ applied 1107 µmol m^−2^ s^−1^ of light intensity to grow *C. vulgaris* in modified OECD medium and observed a specific growth rate of 0.54 d^−1^ and a maximum biomass productivity of 166 mg L^−1^ d^−1^. The authors also reported removal rates ranging from 3.8 to 5.4 mg L^−1^ d^−1^ for NO_3_-N and 1.04 and 1.40 mg L^−1^ d^−1^ for PO_4_-P. Sousa et al.^18^ also used a modified OECD medium and light intensity of 201 µmol m^−2^ s^−1^ to grow *C. vulgaris* and achieved specific growth rates of 0.27–0.54 d^−1^, NO_3_-N removal rate of 2.69 mg L^−1^ d^−1^ and PO_4_-P removal rate of 0.93 mg L^−1^ d^−1^. Overall, the results obtained in this study are consistent with the literature reported under similar conditions, highlighting the suitability of the selected cultivation conditions. El-fayoumy et al.^28^ grew *C. vulgaris* in BG-11 medium under a light intensity of 40 µmol m^−2^ s^−1^ and reported a biomass productivity of only 25 mg L^−1^ d^−1^ after 25 d. Whereas, Yun et al.^29^ cultivated *C. vulgaris* in BG-11 medium under a light intensity of 60 µmol m^−2^ s^−1^ and achieved a biomass productivity of approximately 20 mg L^−1^ d^−1^ after 15 d. These values are significantly lower than those obtained in the present study, likely due to the much lower light intensity used. In sum, the results of the present study show that in this first stage, the microalgae were able to grow well and effectively and reached a biomass concentration and productivity optimum to apply a stressor.

### Growth and productivity under salinity stress

The microalgal growth and nutrient uptake were also analysed during the second stage (stress phase). The presence of salt in the medium can affect the growth and nutrient consumption of the microalgae. It is possible to observe that in stress group 1, the presence of salinity was beneficial for microalgal growth. Table 1 presents the inhibition percentage of each salinity concentration used. In S150 and S300 assays, this value is negative, which means that salinity levels as high as 300 mM did not produce inhibitory effects. The stress phase began with a biomass concentration of 636 ± 15 mg L^−1^ (control 1), 643 ± 15 mg L^−1^ (S150) and 638 ± 27 mg L^−1^ (S300). The ${X}_{max}$ was statistically higher (*p* < 0.05) in S150, followed by S300 and control 1. The ${X}_{max}$ was achieved on the fourth day of the stress phase in control 1 and the seventh day in S150 and S300 assays. Since microalgae are photosynthetic microorganisms, it is important to evaluate the ${F}_{v}/{F}_{m}$ values. This parameter can be used to evaluate the physiological condition of the culture and serve as an indicator of potential exposure to unfavourable conditions^30^. The initial ${F}_{v}/{F}_{m}$ value was similar for all the assays (0.446 ± 0.004). On the last day of the experiment, the ${F}_{v}/{F}_{m}$ value decreased by 43% in control 1, 6% in S150 and 18% in S300. The presence of salt in the culture medium entails some advantages, one of them being the reduction in the risk of microbial contamination, providing a healthier environment for the microalgae to grow in^5^. Maneechote and Cheirsilp^14^ also observed that a 0.5% (approximately 86 mM NaCl) salt concentration led to higher growth in *Scenedesmus* sp.. According to Figler et al.^31^, *C. vulgaris* exhibits moderate tolerance to salinity, thriving in mediums containing up to 20 g L^−1^ NaCl (approximately 342 mM NaCl). Salinity levels above this value may lead to growth inhibition. In the present study, the microalga was only able to grow up to 300 mM, which corresponds to approximately 17.5 g L^−1^ NaCl. Growth inhibition was observed in NaCl concentrations of 450 and 600 mM, which correspond to approximately 26.3 and 35.0 g L^−1^ NaCl, respectively. The nutrient removal rates decreased with increasing salinity for both nutrients tested. The microalgae showed greater NO_3_-N and PO_4_-P uptake in control 1 compared to S150 and S300; that is, the microalgal nutrient uptake ability is affected by the salinity of the medium. The results also suggest that the salinity had a greater impact on NO_3_-N uptake than PO_4_-P uptake. The presence of salt in the medium can disrupt the nutrient adsorption mechanisms, interfering with nutrient uptake. Similar findings were reported for *C. vulgaris* with increasing salinity^31,32^. Regarding stress group 2, the microalgae did not benefit from the salinity levels in the medium. In fact, the salinity concentrations of 450 mM and 600 mM led to cell death. Only after 1 day of stress, the biomass concentration decreases by 20–22% and continues decreasing compared with the initial biomass of the stress phase. Moreover, the inhibition percentage of the assays S450 and S600 showed a high inhibition effect compared with the control 2 assay. The excess of salt in the medium can lead to the collapse of structures in photosystem II, decreasing the assimilation of light and negatively affecting their growth^3^. The reduction of microalgal growth under high salinity can also be due to changes in the osmotic potential. The cell osmotic equilibrium is compromised, potentially resulting in cellular water loss. Since *C. vulgaris* is a freshwater species, this phenomenon can be especially damaging, compromising the cell membrane^32^. Overall, these results indicate that the presence of 150 mM NaCl in the culture medium of *C. vulgaris* can promote higher biomass accumulation and maintain the removal of nutrients compared to the absence of salt in the medium.

**Table 1:** Effect of salinity on biomass concentration, inhibition percentage and nutrient removal rates.

Stress group	Assay	${\text{X}}_{\text{max}}$ (mg L^−1^)	$\text{IP}$ (%)	${\text{RR}}_{{\text{NO}}_{3}-\text{N}} ($mg L^−1^ d^−1^)	${\text{RR}}_{{\text{PO}}_{4}-\text{P}}$ (mg L^−1^ d^−1^)
1	Control 1	904 ± 13^a^	–	6.8 ± 0.2^a^	1.37 ± 0.01^a^
	S150	978 ± 11^b^	− 33.7 (day 7)	4.7 ± 0.5^b^	1.354 ± 0.001^a^
	S300	943 ± 11^c^	− 28.8 (day 7)	1.33 ± 0.02^c^	0.75 ± 0.04^b^
2	Control 2	547 ± 10^a^	–	4.0 ± 0.1^a^	0.35 ± 0.01
	S450	457 ± 18^b^	50.2 (day 4)	1.42 ± 0.06^b^	0*
	S600	408 ± 25^c^	39.8 (day 2)	1.6 ± 0.2^b^	0*

*No PO_4_-P removal was observed.

The numbers that share the same letter (a, b, and c) are not statistically different from one another (*p* > 0.05).

### Biomass biochemical composition under salinity stress

**Fig. 1:**
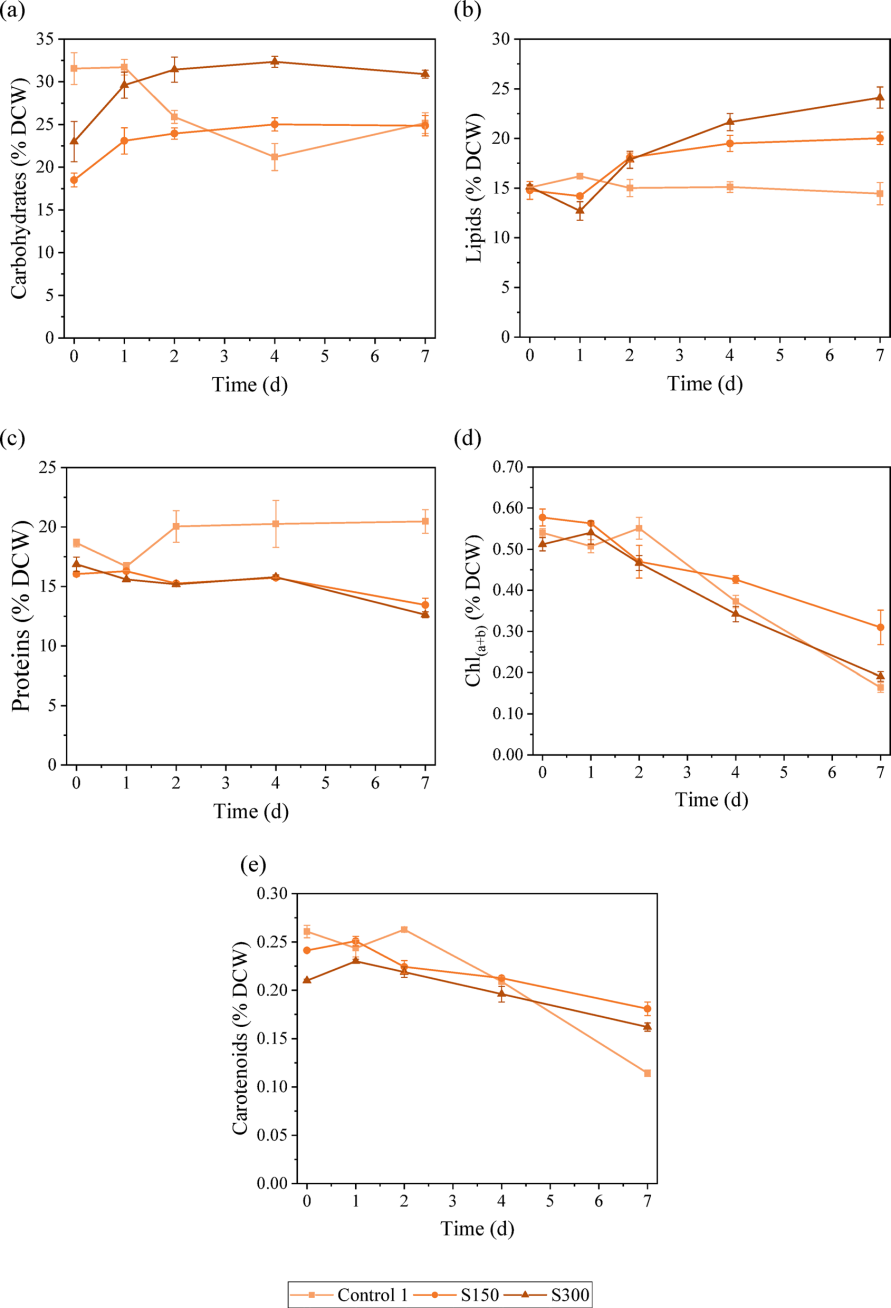
Biomass biochemical composition: (**a**) carbohydrate content, (**b**) lipid content, (**c**) protein content, (**d**) chlorophyll a + b content and (**e**) carotenoid content under different salinity levels.

Analysing the biomass composition helps determine the potential of microalgae for various biotechnological applications. Understanding how salinity stress influences biomass composition is crucial for optimising microalgal cultivation strategies and effectively exploiting their potential. Changes in the biomass composition of *C. vulgaris* were analysed in terms of carbohydrates, lipids, proteins and pigments, which are important indicators of their metabolic state, nutritional value and biofuel potential. The stress responses of this species under different levels of salinity were also discussed. As mentioned before, in the assays S450 and S600, the microalgae did not tolerate the high salinity stress, and cell death occurred. Therefore, the biochemical analysis of these assays is not shown. Figure 1 presents the variation through time of the different compounds studied. A contour graph was also elaborated to illustrate the variation of the studied compounds’ contents with salinity and exposure time (see Figure S1 in Supplementary Information). Regarding the assay S150, it is possible to observe a continuous increase in carbohydrate content with increasing exposure time. The highest values were achieved on day 4 (25.0% ± 0.8% DCW) and day 7 (25% ± 1% DCW), which represents an increase of approximately 35% compared to the initial value (day 0). The lipid content did not change significantly between day 0 and day 1; however, after day 1, lipid accumulation started, reaching the maximum value (20.0% ± 0.6% DCW) on day 7, representing a 36% increase compared to the initial value. Srivastava and Goud^33^ also reported a 56% increase in lipid content in *Chlorella*, reaching 19.6% of DCW, when the NaCl concentration was raised from 5 to 25 mM. The variation of protein content throughout time was minimal, from day 0 until day 4; the protein only decreased 2%. It was possible to notice on the last day of stress a slight drop (15% compared to day 4 and 16% compared with the initial value) in protein content, reaching 13.4% ± 0.6% DCW. The maximum value of protein content (16.3% ± 0.1% DCW) was achieved on day 1. The chlorophyll content followed a downward trend with a − 47% variation between the initial and final values. The highest value, 0.58% ± 0.02% DCW, was achieved on day 0. The decrease in chlorophyll content usually happens when microalgae are under oxidative stress, in this case, due to salinity stress^34^. The carotenoid content followed the same trend with a variation of − 25%. The highest values were observed on day 0 (0.241% ± 0.002% DCW) and day 1 (0.257% ± 0.003% DCW). Overall, it is possible to see an increase in the accumulation of energy storage compounds (carbohydrates and lipids) and a decrease in proteins and pigments.

**Fig. 2:**
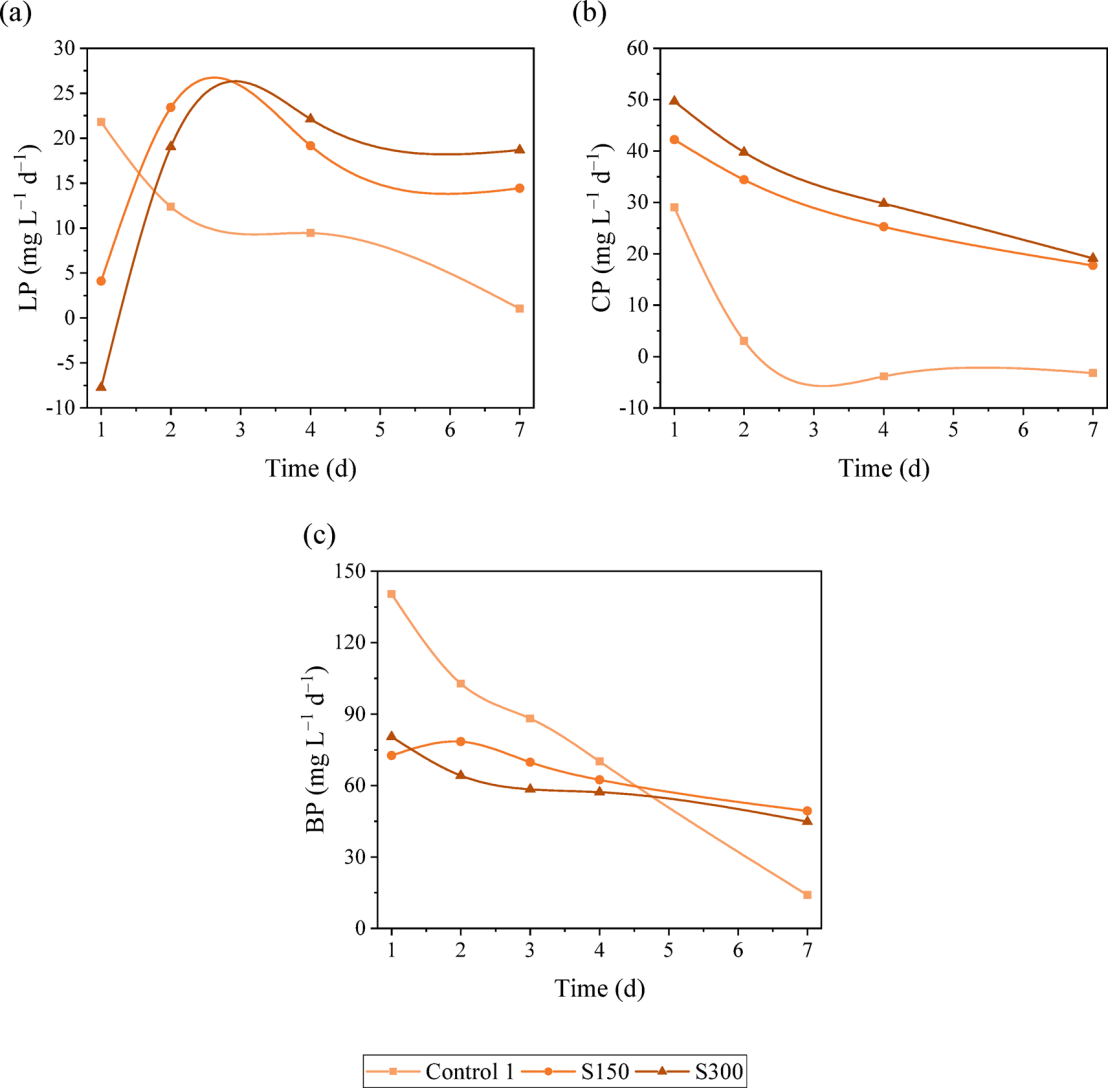
Temporal variability of: (**a**) lipid productivity, (**b**) carbohydrate productivity, and (**c**) biomass productivity under different salinity levels.

Regarding the S300 assay, it is possible to observe that the production of carbohydrates was boosted with the presence of salt. With only 1 day of salt exposure, the carbohydrate content increased by 20%. The maximum value (32.3% ± 0.6% DCW) was achieved on day 4, and it represents a 31% boost in carbohydrate accumulation compared with the initial value. The lipid content slightly decreased on day 1, and from that point, the lipid accumulation was enhanced by 90%, reaching values of 24% ± 1% DCW on day 7. Yun et al.^29^ achieved the highest lipid content (24.5% DCW) with 500 mM NaCl for 2 days. Gour et al.^13^ also observed an 18–23% increase in lipid content of *Chlorella* sp. under 160 mM NaCl, reaching values between 21.7 and 32.2% DCW, depending on whether BG-11 or CHU-10 medium was used. The protein content variation followed the same trend as in the S150 assay. The protein content slightly decreased (7%) until day 4, and then between day 4 and day 7, a greater reduction was observed (20%), reaching the minimum value of 12.6% ± 0.3% DCW. Yun et al.^29^ also observed a decreasing trend in the protein content of *C. vulgaris* with increasing salinity. From Fig. 1, it is possible to observe that the chlorophylls were greatly affected by the presence of salt in the medium. The chlorophyll content slightly increased on the first day, reaching 0.54% ± 0.03% DCW; however, it decreased by 65% from day 1 to day 7, reaching values of 0.19% ± 0.01% DCW. These results support the idea that under high salinity, the proteins involved in photosynthesis and the chlorophylls are damaged and degraded^4,35^. The carotenoid content did not significantly vary until day 4. A slight drop (19%) is observed from day 4 to day 7, reaching the lowest values of carotenoid content (0.162% ± 0.004% DCW). In both assays with the presence of salt, there is a noticeable rise in the accumulation of energy storage compounds alongside a reduction in protein and pigments. Commonly, under stress conditions, in this case, salinity stress (light stress can also be considered since the intensity used in this study was high), the carbon flux can change from protein synthesis towards lipid and carbohydrate production^36,37^.Comparing the control assay with the salinity assays, it is possible to see that in the control assay, the energy storage compounds content decreased throughout time and the protein content increased, whereas in the assays with the presence of salt, an opposite trend is evident. With the increase of the salinity in the medium, microalgae can switch the carbon flux from protein and accumulation to carbohydrate and mainly lipid production to store energy for survival^4,38^. On day 4, the carbohydrate content reached a peak in S150 and S300 assays. Compared with the control, the carbohydrate accumulation was enhanced by 18% and 53% with the presence of 150 mM NaCl and 300 mM NaCl, respectively. Moreover, the highest lipid contents were achieved on day 7 in the S150 and S300 assays. In comparison to the control assay, the lipid accumulation was boosted by 39% and 67% in the assays with 150 mM NaCl and 300 mM NaCl, respectively. This increase in lipid content can be seen as a protective measure against salt-induced damage and as an adaptation response to changes in the osmotic conditions in the medium^14^. Moreover, the salinity stress is known for increasing ROS production, leading to oxidative stress as well as lipid accumulation^7,39^. Farkas et al.^40^ observed an increase in lipid production in *Chlorella* sp. under high salt conditions (0.65 M NaCl), justifying the results on the grounds that the cell membrane and the microalgae’s metabolism may undergo changes in response to osmotic stress. Teh et al.^34^ also stated that the highest lipid content was achieved with F2 medium and artificial seawater at a salinity level of 15 parts per thousand (ppt) in *C. vulgaris* and that the lipid accumulation was triggered to protect the cell against a reduction in volume induced by osmosis. Whereas, Haris et al.^41^ reported that the highest lipid content was observed at 24 ppt in *C. vulgaris* and in *Tetraselmis chuii*. The author also stated that the highest protein content was achieved at 0 ppt (*C. vulgaris*) and 10 ppt (*T. chuii*). El-fayoumy et al.^28^ reported that the accumulation of lipids in *C. vulgaris* was improved by 41% with the presence of 15 g L^−1^ NaCl (approximately 250 mM NaCl) compared to the control (zero salt). However, the carbohydrate and protein content decreased by 22% and 12%, respectively. The authors attributed the decrease in carbohydrate content to a metabolic shift from carbohydrate to lipid synthesis, as evidenced by the concurrent increase in lipid content. By analysing Fig. 2, it is possible to see that both lipid and carbohydrate productivities decrease over time in the control assay. Regarding the lipid productivity in S150 and S300, the maximum quantified values were obtained on day 2 (23.4 mg L^−1^ d^−1^) and day 4 (22.1 mg L^−1^ d^−1^), respectively. However, as mentioned before, the lipid content in both assays was maximum on day 7. The lipid productivity takes the lipid content into account as well as biomass productivity, therefore, the day with high lipid content does not always correspond to the day where the highest lipid productivity was achieved. Based on the trend presented in Fig. 2, the highest value of lipid productivity in S150 and S300 would be obtained approximately on day 3. It is possible to observe that the carbohydrate productivity of S150 and S300 assays followed a downward trend, achieving the highest values on day 1 (42.2 and 46.7 mg L^−1^ d^−1^, respectively). As happened in lipid productivity, the highest values of carbohydrate content did not correspond to the higher carbohydrate contents, which were achieved on day 4 in both assays. Higher lipid productivity is achieved in two-stage cultivation since, in the first stage, the goal is to obtain high biomass productivity^7^.

Comparing the salinity assays, the S150 assay achieved higher lipid productivity, whereas the S300 assay achieved higher carbohydrate productivity. Moreover, the highest value of lipid productivity in S150 was obtained on day 2, implying that the most cost-effective stress phase time could be just 4 days to produce lipids. Whereas, if the aim was to produce carbohydrates, the ideal stress time would be 1 day with 300 mM NaCl. On the other hand, the protein content was the highest on the last day of the control assay, and the salt in the medium led to a 34% and 38% decrease in protein content in the S150 and S300 assays. According to Haris et al.^41^, high salinity levels could negatively affect ATP production by inhibiting the activation of ATP synthase (an enzyme involved in ATP production). Since ATP is crucial for multiple cellular processes, namely protein synthesis, high salt concentration can lead to a decrease in protein content. In the control assay, the microalgal growth began to decrease after day 3 of the second stage. The chlorophyll content was constant up to day 2, and then the chlorophyll content decreased, reaching lower values than those reported in S150 and S300 assays. As mentioned before, the presence of salt (< 300 mM) had a positive effect on microalgal growth. Chlorophyll is intrinsically related to microalgal growth and photosynthetic activity. The carotenoid content followed the same trend as the chlorophyll content.

Overall, these findings suggest that incorporating 300 mM NaCl into the culture medium of *C. vulgaris* can enhance lipid and carbohydrate accumulation. These two compounds are important feedstock for biofuel production. The presence of 150 mM NaCl in the medium can boost lipid productivity in a short period of time (2–3 days) compared to the absence of salt, and the highest carbohydrate productivity was achieved after 1 day of stress with 300 mM NaCl. Moreover, the protein content was negatively affected by the addition of salt in the medium. The salt condition of 150 mM NaCl was the one with the best results regarding pigment content, although it decreased in all the assays.

### Principal component analysis

PCA is a valuable statistical tool commonly used to identify relationships and patterns in complex data sets by reducing dimensionality and preserving the maximum amount of information possible. In this study, PCA was applied to provide insights into the mechanisms underlying microalgal adaptation and physiological responses to different salinity concentrations and exposure times. Table 2 presents the variables analysed and the rotated factor loadings of each PC.

**Table 2:** Rotated factor loadings of each variable to a specific PC.

Variables	PC1	PC2	PC3
Exposure time (d)	**− 0.926**	0.159	0.198
Salinity (mmol)	− 0.005	**− 0.843**	− 0.053
Proteins (% DCW)	0.150	**0.898**	0.038
Lipids (% DCW)	**− 0.744**	*− 0.408*	0.253
Carbohydrates (% DCW)	− 0.063	− 0.057	**− 0.967**
Chlorophylls (% DCW)	**0.927**	0.064	0.320
Carotenoids (% DCW)	**0.881**	0.214	0.294
Eigenvalue	3.31	1.57	1.19
Variance (%)	47.3	22.4	16.9
Cumulative variance (%)	47.3	69.7	86.6

The values in bold represent a strong relationship with the corresponding PC. The value in italics refers to a moderate relationship with the corresponding PC.

PCA correlated different variables related to the biomass biochemical composition, salinity levels and exposure time. Three PCs (corresponding to the total variance of 86.6%) were selected based on the Kaiser criterion. PC1 was positively influenced by variables related to chlorophylls and carotenoids and negatively influenced by the exposure time and lipid content. The salinity, protein and carbohydrate contents have minimal influence on this PC. The high values of eigenvalue (3.31) and variance (47.3%) indicate that PC1 captures nearly half of the total variability observed in the dataset. Whereas the loadings of PC2 showed the relative importance of each variable in contributing to the observed changes in the microalgal responses to the salinity stress, highlighting the contributions of salinity and protein and lipid contents in triggering these responses. The PC3 was mainly driven by variations in carbohydrate content, with lipids and pigment contents also contributing to a lesser extent. PC2 and PC3 contribute to explaining a moderate part of the variability observed in the data, with values of eigenvalue (1.57 for PC2 and 1.19 for PC3) and variance (22.4% for PC2 and 16.9% for PC3) relatively high.

In PC1, it is possible to observe the negative loading of the exposure time (− 0.926) and lipid content (− 0.744), which indicates a strong inverse relationship between these variables and PC1. These results suggest that lower values of PC1 are associated with longer exposure times and high lipid content, also indicating that these two variables follow the same pattern. The high and positive values of the loadings for chlorophyll (0.927) and carotenoid (0.881) contents indicate a strong positive relationship between these variables and PC1, that is, lower values of PC1 are related to lower chlorophyll and carotenoids. It was also possible to conclude that the trend of the variables related to exposure time and lipid content was inverse to that described for chlorophyll and carotenoid content. These results support the findings described above, which are also in line with the literature. As observed before, the lipid content increased with the increase of the exposure time, whereas the pigment contents declined. Lipids are considered long-term energy sources and are accumulated under longer exposure times^42,43^. The pigment content can undergo degradation with increasing exposure time, being that in this study, the light intensity used was high, and the presence of high salinity also led to pigment degradation^4,20,44^. The variation explained by PC2 is strongly and negatively affected by the salinity level (− 0.843) and moderately and negatively affected by the lipid content (− 0.408). Whereas the high and positive loading for protein content (0.898) indicates a strong positive correlation between the protein content and the variability explained by PC2. With these results, it is possible to infer that with the increase of the salinity levels, the lipid content also rises (to a lesser extent), while the protein content decreases. These patterns are in accordance with the findings of the present study and with the literature since proteins are greatly affected by the presence of salt, since ATP production (needed for protein synthesis) can be inhibited^41^. Salinity positively affects lipid content by increasing the production of ROS, leading to oxidative stress and, consequently, lipid accumulation as a protective measure^7,39^. It can be observed that in PC3, the higher value of loading is related to the carbohydrate content. The loading of carbohydrate content was high and negative (− 0.967), indicating an inverse relationship between this variable and PC3. Other variables, such as lipid, chlorophyll and carotenoid contents, have lower loading values (0.253, 0.320 and 0.294, respectively), indicating a moderate positive association between PC3 and these variables. It is possible to state that when the carbohydrate content decreases, a small increase in the lipid and pigment contents can be observed. Carbohydrates and lipids are considered energy storage compounds, and since their production shares precursors, carbohydrates can be used to produce lipids and vice versa^36^.

### Multiple linear regression

MLR was applied to better understand and quantify the impact of salt concentration and exposure time on the content of the studied biomass compounds. By examining the regression coefficients of each independent variable, it is possible to identify key factors that influence the accumulation of biomass compounds. Table 3 presents the regression coefficients of each multiple linear regression model and the corresponding coefficient errors. In the MLR obtained for protein content, it can be observed that the coefficient for exposure time (*β*_*1*_) was not statistically significant, suggesting that there was no linear effect of the exposure time on the protein content. The coefficient for salinity level (*β*_*2*_) obtained was − 4, indicating that an increase in salinity can lead to a decrease in protein content. This result supports the findings of this study and other studies^41^. The squared term coefficient for exposure time (*β*_*11*_) indicates that there was no quadratic effect of this variable; however, the squared term for salinity (*β*_*22*_) was 3, suggesting a positive quadratic effect of this variable on protein content. The interaction term coefficient between exposure time and salinity (*β*_*12*_) was − 1.1, implying that there was a negative interaction effect on protein content. This result also suggests that the combined effect of these two variables (exposure time and salinity) on protein content was lower than their individual effect. By analysing the regression coefficients obtained for lipid content, it can be seen that the coefficient for exposure time (*β*_*1*_) and the coefficient for salinity (*β*_*2*_) were not statistically significant, meaning that there was no linear relationship between these variables individually with lipid content. Moreover, it suggests that neither the linear effect of exposure time nor the linear effect of salinity levels alone significantly influenced the lipid accumulation in the biomass. The squared term coefficients (*β*_*11*_ and *β*_*22*_) were also not statistically significant, meaning that there was no quadratic relationship between the variables and the lipid content. The interaction term coefficient between exposure time and salinity (*β*_*12*_) obtained was 2.9, showing a significant interaction between the combined effect of these variables and the lipid content in the biomass. These results show that with increasing salinity and exposure time, lipid accumulation is enhanced. This trend is observed in the findings of this study and the literature since a stress response to salinity stress and light stress is lipid accumulation^7,14,39,40^. In the MLR model for chlorophyll content, the negative coefficient for exposure time (*β*_*1*_) reveals that with the increase in exposure time, the chlorophyll content decreases. The same trend was observed in the PCA, where the trend of the exposure time was inverse to that described for chlorophyll. The other coefficients in this model were not statistically significant, showing that salinity levels and the interaction between salinity and exposure time did not have significant linear effects on the chlorophyll content. Regarding the MLR model for carotenoids, it is possible to see that the coefficients for exposure time (*β*_*1*_) and for salinity (*β*_*2*_) were not statistically significant, indicating that there was no linear correlation between carotenoid content and these variables alone. The coefficients for the squared terms of exposure time (*β*_*11*_) and salinity (*β*_*22*_) were both negative, suggesting a concave relationship. The interaction term coefficient (*β*_*12*_) was 0.02, implying that the combined effect of salinity and exposure time may be synergistic on the carotenoid content. The *R*^*2*^ values determined for each MLR model were between 0.822 and 0.890, evidencing that the models provide a good fit to the data and accurately predict the content of the chosen biomass compound.

**Table 3:** Regression coefficients of each multiple linear regression model.

	Protein content	Lipid content	Carbohydrate content	Chlorophyll content	Carotenoid content
Regression coefficients					
β_0_	16.6	16.9	27	0.43	0.215
β_1_	–	–	–	− 0.12	–
β_2_	− 4	–	–	–	–
β_1,2_	− 1.1	2.9	–	–	0.02
β_1,1_	–	–	–	–	− 0.05
β_2,2_	3	-	–	–	− 0.02
Coefficient errors					
δ_0_	0.6	0.8	2	0.03	0.009
δ_1_	–	–	–	0.03	–
δ_2_	2	–	–	–	–
δ_1,2_	0.8	0.8	–	–	0.01
δ_1,1_	–	–	–	–	0.01
δ_2,2_	2	–	–	–	0.01
R^2^	0.822	0.836	–	0.844	0.890

The values presented in the table are statistically significant (*α* < 0.05).

## Challenges and future work

Since different microalgal species present diverse tolerance to salinity stress, and this stress is species-dependent, it is also important to perform further research across different microalgae species to select which ones have high salinity tolerance and high compound accumulation capabilities. Future work should also focus on genetic modification to enhance the salt tolerance in microalgae to ensure high biomass productivity and high lipid and/or carbohydrate accumulation; however, the environmental threats posed by the development of genetically modified microalgae must be taken into account. Another way to promote higher resilience and productivity may involve the implementation of adaptive evolution techniques, such as stepwise salinity stress, to gradually acclimate the microalgae. Further research should be done to evaluate whether coupling more stress factors, such as nutrient availability or temperature, can enhance compound productivity. This study was limited by the scale of cultivation (laboratory scale). Scaling up this type of experience is important to validate the data achieved in the present study. However, scaling up might present additional challenges, for example, maintaining consistent salinity levels and ensuring biomass composition consistency. To avoid the use of saline media and increase the cultivation costs, salt water can be added to the culture, avoiding the use of freshwater sources. Future research should focus on cost analysis and the economic viability of these types of processes. In addition to economic considerations, the environmental impact of this process should be assessed through a life cycle assessment, for example.

## Conclusion

In this study, *C. vulgaris* was grown in a two-stage cultivation. By concentrating the biomass, the two-stage cultivation enhanced the microalgae’s ability to respond to the salinity stress, making it a more efficient approach. During the stress phase, different salt concentrations were applied. The microalgae did not tolerate salinity levels above 300 mM. The presence of 150 mM NaCl promoted higher biomass accumulation compared to the absence of salt in the medium. Regarding biomass composition, the addition of 300 mM NaCl enhanced lipid and carbohydrate accumulation. Moreover, the presence of 150 and 300 mM NaCl in the medium led to higher lipid productivity in only 2 days and higher carbohydrate productivity after 1 day of stress, respectively. Therefore, applying salinity stress to this species in a two-stage system can increase its potential as a feedstock for biofuel production.

## Supplementary Information

Supplementary Information.

## Data Availability

All data generated or analysed during this study are included in this published article and its supplementary information files.

## References

[1] Ali, H. E. A., El-fayoumy, E. A., Rasmy, W. E., Soliman, R. M. & Abdullah, M. A. Two-stage cultivation of *Chlorella vulgaris* using light and salt stress conditions for simultaneous production of lipid, carotenoids, and antioxidants. *J. Appl. Phycol.* **33**, 227–239 (2021).

[2] Wu, W. et al. Advancements on process regulation for microalgae-based carbon neutrality and biodiesel production. *Renew. Sustain. Energy Rev.* **171**, 112969 (2023).

[3] Oslan, S. N. H. et al. A review on *Haematococcus pluvialis* bioprocess optimization of green and red stage culture conditions for the production of natural astaxanthin. *Biomolecules* **11**, 256 (2021). 33578851 10.3390/biom11020256 PMC7916564

[4] Santhakumaran, P., Kookal, S. K., Mathew, L. & Ray, J. G. Experimental evaluation of the culture parameters for optimum yield of lipids and other nutraceutically valuable compounds in *Chloroidium saccharophillum* (Kruger) comb. Nov. *Renew. Energy* **147**, 1082–1097 (2020).

[5] Rammuni, M., Ariyadasa, T. U., Nimarshana, P. & Attalage, R. Comparative assessment on the extraction of carotenoids from microalgal sources: Astaxanthin from *H. pluvialis* and β-carotene from *D. salina*. *Food Chem.* **277**, 128–134 (2019). 30502128 10.1016/j.foodchem.2018.10.066

[6] Zhao, T. et al. Enhancement of lipid productivity in *Chlorella pyrenoidosa* by collecting cells at the maximum cell number in a two-stage culture strategy. *Algal Res.* **55**, 102278 (2021).

[7] Aziz, M. M. A. et al. Two-stage cultivation strategy for simultaneous increases in growth rate and lipid content of microalgae: A review. *Renew. Sustain. Energy Rev.* **119**, 109621 (2020).

[8] Nezafatian, E. et al. Enhanced production of bioactive compounds from marine microalgae *Tetraselmis tetrathele* under salinity and light stresses: A two-stage cultivation strategy. *Biores. Technol.* **376**, 128899 (2023). 10.1016/j.biortech.2023.128899 36933578

[9] Li, Q., You, J., Qiao, T., Zhong, D.-B. & Yu, X. Sodium chloride stimulates the biomass and astaxanthin production by *Haematococcus pluvialis* via a two-stage cultivation strategy. *Biores. Technol.* **344**, 126214 (2022). 10.1016/j.biortech.2021.126214 34715336

[10] Arguelles, E. D. & Martinez-Goss, M. R. Lipid accumulation and profiling in microalgae *Chlorolobion* sp. (BIOTECH 4031) and *Chlorella* sp. (BIOTECH 4026) during nitrogen starvation for biodiesel production. *J. Appl. Phycol.* **33**, 1–11 (2021).

[11] Dong, L., Li, D. & Li, C. Characteristics of lipid biosynthesis of *Chlorella pyrenoidosa* under stress conditions. *Bioprocess Biosyst. Eng.* **43**, 877–884 (2020). 31955255 10.1007/s00449-020-02284-x

[12] Almutairi, A. W. Effects of nitrogen and phosphorus limitations on fatty acid methyl esters and fuel properties of *Dunaliella salina*. *Environ. Sci. Pollut. Res.* **27**, 32296–32303 (2020). 10.1007/s11356-020-08531-8 32242318

[13] Gour, R. S., Garlapati, V. K. & Kant, A. Effect of salinity stress on lipid accumulation in Scenedesmus sp. and *Chlorella* sp.: Feasibility of stepwise culturing. *Curr. Microbiol.* 1–7 (2020). 10.1007/s00284-019-01860-z 31925512

[14] Maneechote, W. & Cheirsilp, B. Stepwise-incremental physicochemical factors induced acclimation and tolerance in oleaginous microalgae to crucial outdoor stresses and improved properties as biodiesel feedstocks. *Biores. Technol.* **328**, 124850 (2021). 10.1016/j.biortech.2021.124850 33611021

[15] You, Z., Zhang, Q., Peng, Z. & Miao, X. Lipid droplets mediate salt stress tolerance in *Parachlorella kessleri*. *Plant Physiol.* **181**, 510–526 (2019). 31341003 10.1104/pp.19.00666 PMC6776852

[16] Zhang, C., Hasunuma, T., Lam, S. S., Kondo, A. & Ho, S.-H. Salinity-induced microalgal-based mariculture wastewater treatment combined with biodiesel production. *Biores. Technol.* **340**, 125638 (2021). 10.1016/j.biortech.2021.125638 34358989

[17] Ajayan, K. V., Harilal, C. C. & Preejamol, P. NaCl stress mediated lipid and carotenoid production in freshwater microalga *Kirchneriella obesa* by optimization of medium composition using response surface methodology. *Biofuels* **14**, 883–894 (2023).

[18] Sousa, S., Esteves, A., Salgado, E. & Pires, J. Enhancing urban wastewater treatment: *Chlorella vulgaris* performance in tertiary treatment and the impact of anaerobic digestate addition. *Environ. Technol. Innov.* **34**, 103601 (2024).

[19] Collos, Y. et al. An optical method for the rapid measurement of micromolar concentrations of nitrate in marine phytoplankton cultures. *J. Appl. Phycol.* **11**, 179–184. 10.1023/A:1008046023487 (1999).

[20] Esteves, A. F., Salgado, E. M., Vilar, V. J., Gonçalves, A. L. & Pires, J. C. A growth phase analysis on the influence of light intensity on microalgal stress and potential biofuel production. *Energy Convers. Manag.* **311**, 118511 (2024).

[21] Bligh, E. G. & Dyer, W. J. A rapid method of total lipid extraction and purification. *Can. J. Biochem. Physiol.* **37**, 911–917. 10.1139/o59-099 (1959). 13671378 10.1139/o59-099

[22] Dubois, M., Gilles, K. A., Hamilton, J. K., Rebers, P. T. & Smith, F. Colorimetric method for determination of sugars and related substances. *Anal. Chem.* **28**, 350–356. 10.1021/ac60111a017 (1956).

[23] Van Wychen, S. & Laurens, L. M. *Determination of Total Carbohydrates in Algal Biomass: Laboratory Analytical Procedure (LAP)* (National Renewable Energy Lab. (NREL), 2016).

[24] Lowry, O. H., Rosebrough, N. J., Farr, A. L. & Randall, R. J. Protein measurement with the Folin phenol reagent. *J. Biol. Chem.* **193**, 265–275 (1951). 14907713

[25] Lightenthaler, H. Chlorophylls and carotenoids: Pigments of photosynthetic biomembranes. *Methods Enzymol.* **148**, 350–382. 10.1016/0076-6879(87)48036-1 (1987).

[26] Vieira, J. S. et al. Surface water quality assessment of Lis River using multivariate statistical methods. *Water Air Soil Pollut.* **223**, 5549–5561 (2012).

[27] Pires, J. C. M., Martins, F. G., Sousa, S., Alvim-Ferraz, M. C. & Pereira, M. Selection and validation of parameters in multiple linear and principal component regressions. *Environ. Model. Softw.* **23**, 50–55 (2008).

[28] El-fayoumy, E. A. et al. Co-production of high density biomass and high-value compounds via two-stage cultivation of *Chlorella vulgaris* using light intensity and a combination of salt stressors. *Biomass Convers. Biorefinery* **14**, 22673–22686 (2024).

[29] Yun, C.-J., Hwang, K.-O., Han, S.-S. & Ri, H.-G. The effect of salinity stress on the biofuel production potential of freshwater microalgae *Chlorella vulgaris* YH703. *Biomass Bioenergy* **127**, 105277 (2019).

[30] Mkpuma, V. O., Moheimani, N. R. & Ennaceri, H. Commercial paper as a promising carrier for biofilm cultivation of Chlorella sp. for the treatment of anaerobic digestate food effluent (ADFE): Effect on the photosynthetic efficiency. *Sci. Total Environ.* **898**, 165439 (2023). 37437632 10.1016/j.scitotenv.2023.165439

[31] Figler, A. et al. Salt tolerance and desalination abilities of nine common green microalgae isolates. *Water* **11**, 2527 (2019).

[32] Mohseni, A., Fan, L. & Roddick, F. A. Impact of microalgae species and solution salinity on algal treatment of wastewater reverse osmosis concentrate. *Chemosphere* **285**, 131487 (2021). 34273703 10.1016/j.chemosphere.2021.131487

[33] Srivastava, G. & Goud, V. V. Salinity induced lipid production in microalgae and cluster analysis (ICCB 16-BR_047). *Biores. Technol.* **242**, 244–252 (2017). 10.1016/j.biortech.2017.03.175 28390788

[34] Teh, K. Y. et al. Lipid accumulation patterns and role of different fatty acid types towards mitigating salinity fluctuations in *Chlorella vulgaris*. *Sci. Rep.* **11**, 438 (2021). 33432049 10.1038/s41598-020-79950-3 PMC7801682

[35] Li, S. et al. Mechanism study on the regulation of metabolite flux for producing promising bioactive substances in microalgae Desmodesmus sp. YT through salinity stress. *Algal Res.* **64**, 102721 (2022).

[36] Zhu, J., Tan, X., Hafid, H. S. & Wakisaka, M. A novel strategy to promote microalgal growth and lipid productivity by supplementation of lignin related phenolic elicitors. *Fuel* **334**, 126775 (2023).

[37] Assobhi, B. et al. Influence of salinity, nitrogen and phosphorus concentrations on the physiological and biochemical characteristics of two Chlorophyceae isolated from Fez freshwater, Morocco. *Sci. Rep.* **14**, 8259 (2024). 38589560 10.1038/s41598-024-58864-4 PMC11001895

[38] Bibi, F., Jamal, A., Huang, Z., Urynowicz, M. & Ali, M. I. Advancement and role of abiotic stresses in microalgae biorefinery with a focus on lipid production. *Fuel* **316**, 123192 (2022).

[39] Poh, Z. L. et al. The effect of stress environment towards lipid accumulation in microalgae after harvesting. *Renew. Energy* **154**, 1083–1091 (2020).

[40] Farkas, A. et al. Salinity stress provokes diverse physiological responses of eukaryotic unicellular microalgae. *Algal Res.* **73**, 103155 (2023).

[41] Haris, N. et al. Effect of different salinity on the growth performance and proximate composition of isolated indigenous microalgae species. *Aquac. Rep.* **22**, 100925 (2022).

[42] Chen, J. et al. Interaction of Scenedesmus quadricauda and native bacteria in marine biopharmaceutical wastewater for desirable lipid production and wastewater treatment. *Chemosphere* **313**, 137473 (2023). 36481174 10.1016/j.chemosphere.2022.137473

[43] Chia, S. R. et al. Sustainable approaches for algae utilisation in bioenergy production. *Renew. Energy* **129**, 838–852 (2018).

[44] Levasseur, W., Perré, P. & Pozzobon, V. Chlorella vulgaris acclimated cultivation under flashing light: An in-depth investigation under iso-actinic conditions. *Algal Res.* **70**, 102976 (2023).

