# Supplementary material for: Exploring salinity-induced biochemical changes in Chlorella vulgaris using statistical modelling
Source: Sci Rep. 2025 Oct 21;15:36521. doi: 10.1038/s41598-025-11110-x (PMC12541059; doi:10.1038/s41598-025-11110-x)
Supplement: Supplementary file 1 — Supplementary Information. [file 41598_2025_11110_MOESM1_ESM.docx]

**Exploring Salinity-Induced Biochemical Changes in *Chlorella vulgaris* using Statistical Modelling**

Esteves, A.F.^1,2,3^, Gonçalves, A.L.^1,2,4^, Vilar, V.J.P.^2,3^, Pires, J.C.M.^1,2^*

^1^ LEPABE – Laboratory for Process Engineering, Environment, Biotechnology and Energy, Faculty of Engineering, University of Porto, Rua Dr. Roberto Frias, 4200-465 Porto, Portugal.

^2^ ALiCE – Associate Laboratory in Chemical Engineering, Faculty of Engineering, University of Porto, Rua Dr. Roberto Frias, 4200-465 Porto, Portugal.

^3^ LSRE-LCM – Laboratory of Separation and Reaction Engineering-Laboratory of Catalysis and Materials, Faculty of Engineering, University of Porto, Rua Dr. Roberto Frias, 4200-465 Porto, Portugal.

^4^ CITEVE – Technological Centre for the Textile and Clothing Industries of Portugal, Rua Fernando Mesquita, 2785, 4760-034 Vila Nova de Famalicão, Portugal

*Corresponding author

Telephone: +351 22 508 2262

E-mail addresses: jcpires@fe.up.pt (Pires, J.C.M.)

| (a) | (b) |
| --- | --- |
|  |  |
|  |  |
| (c) | (d) |
|  |  |
|  |  |
| (e) |  |
|  | |

**Figure S1.** Contour map representing the variation of different compound content with salinity and exposure time.
